# Screening and brief intervention (SBI) for hazardous alcohol use in an inner-city emergency ward in The Netherlands: three-month follow-up

**DOI:** 10.1186/1940-0640-8-S1-A28

**Published:** 2013-09-04

**Authors:** Angelique van Gaalen, Carla Hagestein de Bruijn, Christien van der Linden

**Affiliations:** 1 Medical Center Haaglanden, Westeinde, The Hague, The Netherlands

## Introduction

Hazardous alcohol use carries elevated risk for future health problems. Visits to an ED offer a chance to promote change in drinking behavior.

## Objective

This is the first prospective, observational pilot study in the Netherlands to examine whether an ED SBI intervention is effective in identifying patients with hazardous alcohol use and in motivating them to reduce their alcohol use.

## Methods

*Subjects:* Of the 41900 consecutive ED patients age 18 years and older who presented at the ED between November 2012 and November 2013, 22537 were screened for hazardous alcohol use. *Measures:* AUDIT-C was used to assess drinking behaviour. Cut-off values were 4 for women and 5 for men. *Intervention:* Patients with elevated AUDIT-C scores were provided educational folders. Brief interventions were performed by ED nurses and physicians, who were trained in motivational interviewing. *Follow-up:* At 3 months, patients with a positive AUDIT-C score were called and recent drinking pattern was assessed.

## Results

10% of ED patients screened had elevated AUDIT-C scores. Of these, 37% received educational material; the proportion offered a brief intervention raised from 7% to 17% in time. Of all ED patients with positive AUDIT-C scores, 33% either lowered or stopped their alcohol use at three month follow-up (p<0.005). Of subjects who received educational material and who were given brief interventions, 52% and 77%, respectively, either lowered or stopped their alcohol intake at three-month follow-up (p<0.005). Kruskall Wallis test showed that differences between groups were significant as well (p<0.005).

## Discussion

In this large, prospective study in The Netherlands, SBI has been demonstrated to be effectively applied in an inner-city ED in The Netherlands. Just screening results in considerable lowering of alcohol intake. Educational folders and motivational interventions have shown to be effective in further reducing alcohol intake.

